# Autophagy‐induced cell death by aqueous and polyphenol‐enriched extracts of honeybush (*Cyclopia* spp.) in liver and colon cancer cells

**DOI:** 10.1002/fsn3.4214

**Published:** 2024-05-15

**Authors:** Sedicka Samodien, Maryna de Kock, Elizabeth Joubert, Dalene de Beer, Jurgen Kriel, Wentzel C. A. Gelderblom, Mariska Lilly

**Affiliations:** ^1^ Applied Microbial and Health Biotechnology Institute Cape Peninsula University of Technology Bellville South Africa; ^2^ Department of Medical Bioscience Program University of Western Cape Bellville South Africa; ^3^ Plant Bioactives Group, Post‐Harvest & Agro‐Processing Technologies Agricultural Research Council, Infruitec‐Nietvoorbij Stellenbosch South Africa; ^4^ Department of Food Science Stellenbosch University Stellenbosch South Africa; ^5^ Central Analytical Facilities, Electron Microscopy Unit Stellenbosch University Stellenbosch South Africa; ^6^ Department of Biochemistry Stellenbosch University Stellenbosch South Africa

**Keywords:** autophagy, cell proliferation, *Cyclopia*, mitochondrial integrity, polyphenols

## Abstract

The anti‐cancer potential of *Cyclopia* species (honeybush) has been demonstrated in several models. The present study investigated the effects of aqueous and polyphenol‐enriched (PE) extracts of *C. subternata* and *C. genistoides*, as well as mangiferin and hesperidin, on different cell growth parameters in human liver (HepG2) and colon (HT‐29) cancer cells. Mangiferin and hesperidin were most abundant in *C. genistoides* and *C. subternata*, respectively. *Cyclopia subternata* extracts had the highest ferric‐reducing antioxidant capacity. Following exposure of the cells to the extracts and compounds, cell viability, proliferation, and death (apoptosis and autophagy) were determined. *Cyclopia subternata* extracts reduced cell viability and inhibited cell proliferation the most, associated with depletion of ATP. In HepG2 cells, the PE extracts were less effective than the aqueous extracts in reducing cell viability but more effective in inhibiting cell proliferation. Despite disrupting cell growth, none of the extracts induced apoptosis. The aqueous extracts affected autophagy in both cancer cells. Disruption of mitochondrial membrane integrity by the different extracts, presumably via polyphenol/iron interactions, is postulated to be involved; however, mangiferin and hesperidin had no effect, suggesting that other polyphenols and/or complex interactions between compounds are likely responsible for the differential cytotoxic and/or cytoprotective effects of the extracts.

## Graphical abstract

Aqueous extracts of honeybush have been found to decrease cell viability and hinder cell proliferation in HepG2 and HT‐29 cells. While they did not affect apoptosis, they induced autophagy in both cancer cell lines. Similarly, honeybush polyphenols, mangiferin, and hesperidin, when in pure form, also diminished cell viability and impeded cell proliferation without affecting apoptosis or autophagy at the tested concentrations. This implies that other polyphenols and/or intricate compound interactions probably play a pivotal role in the varying cytotoxic and/or cytoprotective effects of the extracts.

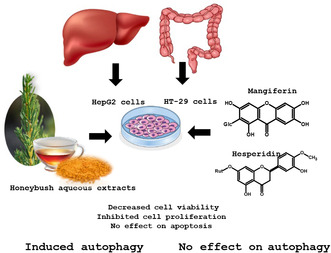

## 1 INTRODUCTION

The antioxidant capabilities of polyphenols, which are found in common food items including fruit, vegetables, and tea, are linked to the prevention of several chronic illnesses and cancer (Dai & Mumper, 2010). These compounds are considered beneficial as they may inhibit cell proliferation, protect against oxidative stress, and modulate cell signaling pathways related to cell survival. Accordingly, the daily consumption of polyphenols as active ingredients in dietary supplements has gained considerable popularity (Cory et al., 2018).

Recently, the anti‐cancer effects of polyphenols via autophagy modulation were reported (Deng et al., 2019; Li et al., 2016; Moosavi et al., 2018; Sun et al., 2019; Vidoni et al., 2020). These effects were due to polyphenols interfering with various pathways, including canonical (Beclin‐1‐dependent), non‐canonical (Beclin‐1‐independent), and other signaling pathways, including the nuclear factor‐κB (NF‐κB)‐related, mitogen‐activated protein kinase/c‐ and Jun N‐terminal kinases pathways (Bishayee & Darvesh, 2012). Two compounds of interest are mangiferin (a xanthone) and hesperidin (a flavanone) because they are abundant in *Cyclopia* extracts, which have been shown to have anti‐cancer properties in different models (Petrova et al., 2011; Sissing et al., 2011). The herbal tea known as honeybush tea is made from the stems and leaves of *Cyclopia* plants (Joubert et al., 2019). Mangiferin was shown to regulate cardiac autophagy in rat cardiomyocytes by suppressing the mammalian target of rapamycin complex 1 in downstream signal transduction (Hou et al., 2018). Hesperidin activated the phosphatidylinositol 3‐kinase/Protein kinase B (Akt)/mTOR pathway, thereby inhibiting excessive autophagy in mice with myocardial ischemia/reperfusion injury (Li et al., 2016). Both compounds exhibit a range of pharmacological properties, including anti‐cancer properties, as previously reviewed (Gold‐Smith et al., 2016; Roohbakhsh et al., 2015). Mangiferin has been demonstrated to reduce inflammation, cause cell cycle arrest, decrease proliferation/ metastasis, promote apoptosis in cancer cells, and protect against deoxynucleic acid (DNA) damage and oxidative stress (Gold‐Smith et al., 2016). Hesperidin was shown to inhibit tumor growth by targeting a number of cellular proteins simultaneously, including caspases, leading to the induction of apoptosis, B‐cell lymphoma 2 (Bcl‐2), Bcl‐2‐associated X protein (Bax), cyclooxygenase‐2 (COX‐2), matrix metalloproteinase‐2 (MMP‐2), and matrix metalloproteinase‐9 (MMP‐9) to inhibit angiogenesis and metastasis, thus interfering at several stages of cancer (Roohbakhsh et al., 2015).

The scarcity of studies focusing on human liver and colon cancer cells and a lack of understanding of how polyphenol‐rich extracts exhibit their anti‐cancer effects through non‐apoptotic cell death, such as autophagy, motivated the present study, utilizing extracts of *C. genistoides* and *C. subternata*. The predominant phenolic compounds in *C. genistoides* belong to the xanthone and benzophenone classes (Beelders et al., 2014). Compounds belonging to these classes are less prominent in *C. subternata*, and its extracts are differentiated by the higher hesperidin levels and the presence of flavone, scolymoside, and dihydrochalcones (De Beer et al., 2012; Yoshida et al., 2020).

It is crucial to clarify the anti‐cancer effects of honeybush extracts given their health‐promoting qualities and prospective usage as dietary supplements or pharmaceuticals. The aim of the study was to evaluate the chemopreventive effects of aqueous and polyphenol‐enriched (PE) extracts of *C. subternata* and *C. genistoides* on different cell survival parameters in human liver and colon cancer cells. Mangiferin and hesperidin were included to evaluate the chemopreventive properties of a single compound versus complex polyphenolic mixtures as represented by the extracts.

## 2 MATERIALS AND METHODS

### 2.1 Materials

Sigma‐Aldrich (St. Louis, MO, USA) supplied Folin–Ciocalteu's phenol reagent, gallic acid, dimethylsulfoxide (DMSO), Trolox, ferrous sulfate heptahydrate (99% FeSO_4_), 2,4,6‐tris(2‐pyridyl)‐*s*‐triazine, mangiferin (>97%), hesperidin (97%), acridine orange (AO), propidium iodide (PI), and Hoechst 33342 (HO), while Merck Chemicals (Darmstadt, Germany) supplied other analytical grade chemicals. Authentic phenolic standards for high‐performance liquid chromatography (HPLC) (>95%) were purchased from Sigma‐Aldrich (hesperidin), Extrasynthese (Genay, France; eriocitrin, luteolin), and Phytolab (Vestenbergsreuth, Germany; mangiferin, vicenin‐2).

Consumables and reagents for cell culture, that is, Dulbecco's Modified Eagle's Medium (DMEM), Dulbecco's Phosphate Buffered Saline (DPBS), Eagle's Minimum Essential Medium (EMEM), fetal bovine serum (FBS), L‐glutamine, trypsin versene, Hank's balanced salt solution, and penicillin/streptomycin/fungizone (Amphotericin B), were obtained from Whitehead Scientific (Braine‐l'Alleud, Belgium). Tissue culture flasks, white 96‐well microtiter plates, and disposable serological pipettes were obtained from Ascendis Medical (Gauteng, South Africa). Black 96‐well microtiter plates, the CellTitre Glo® Luminescent Cell Viability Assay kit, the Caspase‐Glo® 3/7 assay kit, and Reporter lysis buffer 5× concentrated were obtained from Anatech (Southampton, UK). Cell proliferation ELISA, 5‐bromo‐2′‐deoxyuridine (BrdU) chemiluminescent assay kit, and sodium pyruvic acid were purchased from Sigma‐Aldrich (St. Louis, Missouri, USA).

### 2.2 Preparation and characterization of extracts

The PE extracts of green *C. subternata* (PECsub; Raps Foundation, Freising‐Weihenstephan, Germany; lot nr 4448002) and *C. genistoides* (PECgen; Raps Foundation; lot nr A‐he‐40‐a‐02.05.06‐tb) were the same commercial extracts as described previously (Van der Merwe et al., 2017). For the preparation of the aqueous extracts, shoots of *C. genistoides* and *C. subternata* plants were harvested from cultivated plants on commercial farms in the Western Cape, South Africa. The species for seed collected in the wild and used for propagation was originally identified by J.H. de Lange (SANBI Kirstenbosch) based on the classification by Schutte (1997). The shoots were cut and dried to produce the green (unoxidized) herbal teas (Le Roux et al., 2008). Briefly, the plant material (100 g) was steeped in freshly boiled deionized water (1 L) for 30 min at room temperature, followed by filtration and freeze‐drying. The dried, aqueous extracts (ACsub and ACgen) were stored at room temperature in dark amber glass vials under desiccation. Retention samples were coded MRC2008_36 (ACsub) and MRC2008_12 (ACgen).

The PECsub and PECgen extracts were previously characterized in terms of the total polyphenol content, individual polyphenol content, and ferric‐reducing antioxidant power (FRAP) (Van der Merwe et al., 2017). The same assays and procedures were employed for ACsub and ACgen. This entailed using the Folin–Ciocalteu colorimetric and the FRAP assays (Arthur et al., 2011), as well as HPLC‐diodearray detection (DAD) quantification of the individual polyphenols of ACsub (De Beer et al., 2012) and ACgen (Beelders et al., 2014).

Liquid chromatography‐mass spectrometry (LC–MS) analysis of samples was performed on an Acquity ultra‐performance liquid chromatography (UPLC) system coupled to a Synapt G2 Q‐time of flight mass spectrometer (Waters) to tentatively identify phenolic compounds. The UPLC consisted of a binary solvent manager, sample manager, column heating compartment, and DAD. The electrospray ionization source was operated in negative mode. Mass calibration was achieved using sodium formate, and the lockspray was leucine enkephalin. The capillary voltage and sampling cone voltages were −2.5 kV and 15.0 V, respectively. The source and desolvation temperatures were 120 and 275°C, respectively. The desolvation and cone gas (N_2_) flow rates were 650 and 50 L/h, respectively. An *m/z* range of 180–1500 amu was scanned. MS/MS analysis was done using a collision energy of 30 V.

### 2.3 Human cancer cell lines

The human hepatocellular carcinoma cell line (HepG2) and human colorectal adenocarcinoma cell line (HT‐29) were procured from the American Type Culture Collection (ATCC). Cells were cultured as monolayers in EMEM (HepG2) and DMEM (HT‐29) media containing 10% heat‐inactivated FBS and L‐glutamine (2 mM). Non‐essential amino acids (10 mM) and sodium pyruvate (100 mM) were also added to EMEM. Cells were incubated in a humidified atmosphere of 5% CO_2_/95% air at 37°C for 24 h prior to the start of the experiments.

### 2.4 Treatment with extracts and pure polyphenols

The extract and compound (mangiferin and hesperidin) solutions in DMSO were diluted at different concentrations in filter‐sterilized (0.22 μm) culture media containing 0.5% FBS. The final concentration of DMSO did not exceed 1%. Cells were subjected to the different treatment protocols described below, and the IC_50_ (50% inhibition of a specific biological response) was determined. IC_50_ was determined by combining 10 individual data points for two independent experiments using extract concentrations ranging from 0.1 to 2 mg/mL. Both cell lines were seeded at 1 × 10^4^ cells per well in 96‐well microtiter plates. Cells were seeded for 24 h at 37°C and incubated at 5% CO_2_/95% air. Thereafter, the cells were treated for 24 h with the respective extracts or pure compound concentrations as determined. Five replicates of the different dilutions of each extract and polyphenol were tested, and the experiments were repeated in triplicate.

### 2.5 Cell growth parameters

#### 2.5.1 Cell viability

Cell viability was determined based on the quantification of adenosine triphosphate (ATP) (representing metabolically active cells) using the CellTitre Glo® luminescent cell viability assay. The luminescence signal (expressed as relative light units) was determined on a Veritas microplate Luminometer (Turner Biosystems, Madison, USA), and ATP production was expressed as a percentage (%) of the control cells. The IC_50_ values, i.e., the amount of extract (mg/mL) or compound (mM) required for a 50% reduction in cell viability, were determined using the GraphPad Prism Software version 5.04 (GraphPad Software, LaJolla, California, USA).

#### 2.5.2 Cell proliferation

The antiproliferative effects of the extracts and polyphenols were assessed using the Cell Proliferation ELISA and the BrdU chemiluminescent assay. The quantification of DNA synthesis was detected by chemiluminescence (expressed as relative light units) using the Veritas Microplate Luminometer. Data were expressed as a percentage of the control cells. The IC_50_ values were determined as described above.

### 2.6 Modulation of cell death

#### 2.6.1 Apoptosis

Apoptosis was determined with the Caspase‐Glo® 3/7 Assay kit. The extracts were tested at concentrations equal to their IC_50_ values for reduction of cell viability and inhibition of cell proliferation. Mangiferin (1.25 and 0.31 mM) and hesperidin (0.23 and 0.06 mM) were also tested. The highest concentrations of mangiferin and hesperidin that could be tested were limited due to their poor solubility in the incubation medium. Staurosporine (100 nM) dissolved in DMSO was used as the positive control. Analyses were conducted using a Veritas microplate luminometer, and relative light units were used to calculate the fold increase compared to the control treatment regimens. Cell viability using the Cell Titre‐Glo® Luminescence assay was monitored in conjunction with the caspase‐3 assay.

#### 2.6.2 Fluorescent microscopy—Triple staining technique

A triple staining dye method was used to study types of cell death (necrosis, apoptosis, or autophagy) induced after exposure of HepG2 and HT‐29 cells to ACsub and ACgen at concentrations equaling their IC_50_ values for reduction of cell viability and inhibition of cell proliferation. Mangiferin (1.25 and 0.31 mM) and hesperidin (0.23 and 0.06 mM) were also included at the two highest concentrations utilized in the cell viability assay. Cells were seeded (25 × 10^4^ cells/2 mL) in their respective media onto sterilized coverslips in 35 mm Petri dishes and treated with honeybush extracts and pure compounds for 24 h. Cells were exposed to staurosporine (100 nM) as a positive control for apoptosis, while starved cells in nutrient‐deficient conditions (media only in the absence of supplements incubated for 4 h) were included as a positive control for autophagy. The cells were labeled with HO (3.5 μg/mL), AO stain (4.0 μg/mL), and PI (40 μg/mL) in DPBS for 30 and 5 min, respectively, at 37°C. Cells were rinsed twice in DPBS and viewed with a Zeiss inverted Axiovert CFL40 microscope, and the images were captured with a Zeiss Axiovert MRm monochrome camera (Zeiss Gottingen, Germany). Zeiss filters 2, 9, and 15 were used for HO‐, AO‐, and PI‐stained cells, respectively. The magnification of photographs was ×40 and ×100.

#### 2.6.3 Scanning transmission electron microscopy

For verification of the effects of ACgen and ACsub on autophagy during AO staining, HepG2 and HT‐29 cells were seeded as described above and incubated for 24 h with ACsub and ACgen at concentrations equaling their IC_50_ values for reduction in cell viability as described above. Starved cells in nutrient‐deficient conditions (media in the absence of supplements incubated only for 4 h) were included as a positive control for autophagy. Subsequently, cells were fixed for 10 min at room temperature using a mixture of 2.5% glutaraldehyde and 4% formaldehyde in 0.1 M Sorenson's phosphate buffer. Cells were incubated in 2% reduced osmium tetroxide (a mixture of 4% OsO_4_ and 3% potassium ferricyanide, 1:1) for 60 min on ice, followed by a 20‐min incubation with thiocarbohydrazide at room temperature. This was followed by a 30‐min incubation in aqueous osmium tetroxide at room temperature and an overnight incubation in 1% uranyl acetate at 4°C (Tapia et al., 2012). Between each staining incubation, cells were washed three times with deionized water.

After overnight incubation, samples were further incubated with lead aspartate for 30 min at room temperature before dehydration in an ethanol series of increasing concentrations for 5 min each on ice (20%, 50%, 70%, 90%, and 100%, and anhydrous 100%). The anhydrous ethanol step was repeated once for 10 min at room temperature, followed by incubation with an acetone and Epon (Epon 812, Agar Scientific Ltd, Essex, UK, G3759) mixture (1:1) for 1 h at room temperature. The cells were then incubated twice in 100% Epon for 90 min at room temperature. After the removal of the last Epon, a flat‐bottom capsule (Agar Scientific) was filled with 100% Epon and placed upside down on the area marked before. The dish with an inverted coverslip was incubated at 60°C for at least 48 h. Once polymerized, the inverted capsule was broken away from the coverslip with the single cell layer embedded in the Epon in the capsule, with the pattern of the grid clearly visible with a stereomicroscope. After identifying the region of interest, the capsule was trimmed further using a glass knife. The resin block face was then sectioned using a Leica UC7 ultramicrotome system (Leica Microsystems, Austria) and an Ultra 45° 3 mm diamond knife (Diatome US, Hatfield, PA, USA, MS16427), cut into ultrathin sections of 70 nm, and collected onto TEM grids.

Scanning transmission electron microscopy (STEM) was conducted using a ThermoFisher Apreo SEM equipped with a TEM detector. Sections were imaged at 22 kV with an accelerating current of 0.2 nA in HAADF mode at a working distance of 8.3 mm. Images were captured at a scan speed of 2 μs with a pixel resolution of 3840 × 2160.

### 2.7 Statistical analysis

Analysis of variance (ANOVA) was used to test for significant group effects for more than two groups, using the GLM procedure of SAS (Cary, NC, USA). The Tukey–Cramer adjustments were made automatically for unbalanced data. Levene's test was used to test for the homogeneity of the variances, and Tukey's test was used as the post‐hoc test. When only two groups were present, *t*‐tests were used. *p* < .05 was considered statistically significant.

## 3 RESULTS

### 3.1 Phenolic composition and iron‐reducing antioxidant power (FRAP) of extracts

The total polyphenol content of PECsub, determined by the Folin–Ciocalteu assay, was higher than that of the other extracts (Table 1). However, the sum of quantified individual phenolic compounds in the extracts was highest for PECgen (Table 1). The LC–MS data for the phenolic compounds in the extracts of the two *Cyclopia* species are provided in Supplementary information (Tables S1 and S2). The phenolic composition of the extracts clearly differs quantitatively, and qualitatively as shown by the HPLC‐DAD and LC–MS data, notably the much higher xanthone and benzophenone content of *C. genistoides* extracts, as well as the high dihydrochalcone content of the *C. subternata* extracts. The dihydrochalcones were present in low levels in the *C. genistoides* extracts. The flavone, luteolin, the flavanones, eriodictyol‐*O*‐deoxyhexose‐*O*‐hexose and (2*S*)‐5‐*O*‐neohesperidosylnaringenin, and the minor benzophenone, maclurin‐di‐*O,C*‐hexose, were not detected in the *C. subternata* extracts using LC–MS. Isorhoifolin was also detected in the *C. subternata* extracts by LC–MS, but not in the *C. genistoides* extracts. ACsub contained approximately equal quantities of total xanthones, benzophenones, and dihydrochalcones. Their relative ratio was different in PECsub, with this extract containing higher levels of all phenolic compounds than ACsub, except for maclurin‐di‐*O,C*‐hexose. This benzophenone was not detected in either of the *C. subternata* extracts using LC–MS. The two *C. genistoides* extracts had very similar phenolic compositions in terms of the content of the various phenolic groups. The presence of trace quantities of an unidentified dihydroxybenzoic acid‐*O*‐pentose, (2*R*)‐5‐*O*‐neohesperidosylnaringenin (only in ACgen), and scolymoside (only in PECgen) was confirmed in the *C. genistoides* extracts by LC–MS.

**TABLE 1:** Phenolic composition,^a^ total polyphenol content,^b^ and FRAP^c^ of *Cyclopia subternata* and *C. genistoides* extracts.

Polyphenolic subgroup	Compound/ parameter	Substitution	ACsub	PECsub	ACgen	PECgen
Xanthones 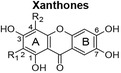	Mangiferin	R_1_ = β‐D‐glucopyranosyl; R_2_ = H	1.70	3.32	6.77	8.35
	Isomangiferin	R_1_ = H; R_2_ = β‐D‐glucopyranosyl	0.59	0.90	2.61	2.59
		Total	2.29	4.22	9.38	10.94
Flavanones 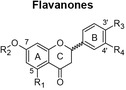	Eriocitrin	R_1_ = R_3_ = R_4_ = OH; R_2_ = rutinosyl	0.38	0.93	0.03	0.09
	Hesperidin	R_1_ = R_3_ = OH; R_2_ = rutinosyl; R_4_ = OCH_3_	0.82	0.88	0.36	0.59
	EDH	R_1_ = R_3_ = R_4_ = OH; position of sugars not known	nd	nd	0.14	0.15
	SNAR	R_1_ = neohesperidosyl; R_2_ = R_4_ = H; R_3_ = OH	nd	nd	0.67	0.38
		Total	1.20	1.81	1.20	1.21
Dihydrochalcones 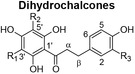	PDG	R_1_ = R_2_ = β‐D‐glucopyranosyl; R_2_ = H	1.80	2.69	0.09	0.18
	HPDG	R_1_ = R_2_ = β‐D‐glucopyranosyl; R_3_ = OH	0.55	0.98	0.23	0.09
		Total	2.35	3.67	0.32	0.27
Flavones 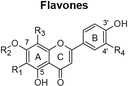						
	Scolymoside	R_1_ = R_3_ = H; R_2_ = rutinosyl; R_4_ = OH	0.73	2.03	nq	nq
	Vicenin‐2	R_1_ = R_3_ = β‐D‐glucopyranosyl; R_2_ = R_4_ = H	0.20	0.22	0.52	0.48
		Total	0.93	2.32	0.52	0.48
Benzophenones 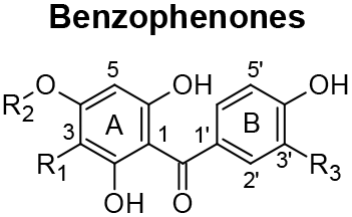	IDG	R_3_ = H; R_1_ = R_2_ = β‐D‐glucopyranosyl	0.91	1.34	2.94	1.79
	IMG	R_2_ = R_3_ = H; R_1_ = β‐D‐glucopyranosyl	0.95	1.43	1.79	2.08
	MMG	R_2_ = H; R_3_ = OH; R_1_ = β‐D‐glucopyranosyl	0.19	0.18	0.58	0.97
	Maclurin‐di‐*O*,*C*‐hexose	R_3_ = OH; position of sugars not known	nd	nd	0.07	0.07
		Total	2.05	2.95	5.38	4.91
	∑Compounds^a^		8.82	14.97	16.80	17.81
	Total polyphenol (g GAE/100 g)^b^		19.5	24.8	20.6	21.9
	FRAP (mmol TE/g)^c^		1.73	1.79	1.60	0.99

*Note*: Values represent the means of duplicate analyses.

Abbreviations: ACgen and PECgen, aqueous and polyphenol‐enriched extracts of *C. genistoides*; ACsub and PECsub, aqueous and polyphenol‐enriched extracts of *C. subternata*; EDH, eriodictyol‐*O*‐deoxyhexose‐*O*‐hexose; GAE, gallic acid equivalents; HPDG, 3′,5′‐di‐β‐D‐glucopyranosyl‐3‐hydroxyphloretin; IDG, 3‐β‐D‐glucopyranosyl‐4‐*O*‐β‐D‐glucopyranosyliriflophenone; IMG, 3‐β‐D‐glucopyranosyliriflophenone; MMG, 3‐β‐D‐glucopyranosylmaclurin; Nd, not detected; Nq, detected by LC–MS, but not quantifiable using HPLC‐DAD; PDG, 3′,5′‐di‐β‐D‐glucopyranosylphloretin; SNAR, (2S)‐5‐*O*‐neohesperidosylnaringenin; TE, Trolox equivalents.

^a^ g/100 g extract.

^b^ Determined using the Folin–Ciocalteu reagent in g gallic acid equivalents/100 g extract.

^c^ mMol Trolox equivalents/g extract.

The FRAP values of the *C. subternata* extracts were higher than those of the ACgen extract, with PECgen having the lowest value (Table 1).

### 3.2 Modulation of cell viability

**FIGURE 1:**
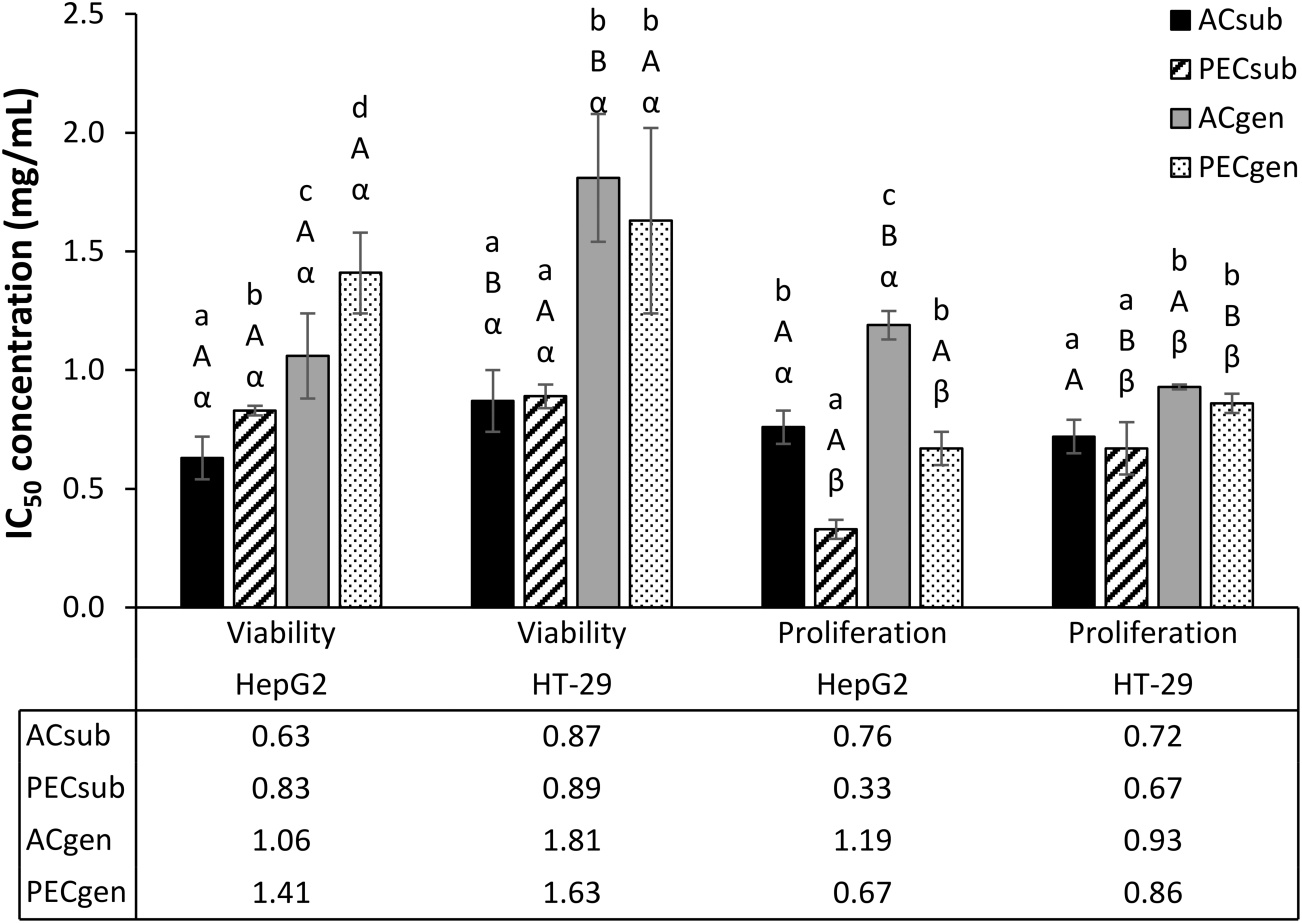
IC_50_ concentrations (mg/mL) of *Cyclopia subternata* and *C. genistoides* extracts concerning the reduction in cell viability (% ATP production) and the inhibition of cell proliferation (BrdU incorporation) in human liver (HepG2) and colon (HT‐29) cancer cells. The mean values ± standard deviations obtained from five replications of at least two independent experiments are presented. Statistical significance (*p* < .05) between extracts for each parameter is denoted by distinct lowercase letters. Furthermore, variations (*p* < .05) observed between the different cell lines for each parameter are represented using distinct uppercase letters. Significant differences (*p* < .05) between the reduction in cell viability and the inhibition of cell proliferation are indicated by the utilization of Greek letters. IC_50_ is the concentration yielding 50% inhibition. ACsub and PECsub refer to the aqueous and polyphenol‐enriched extracts of *C. subternata*, respectively. Similarly, ACgen and PECgen denote the aqueous and polyphenol‐enriched extracts of *C. genistoides*, respectively.

Figure 1 summarizes the IC_50_ values of the extracts for the reduction of cell viability. In HepG2 cells, the aqueous extract of each *Cyclopia* species elicited a greater response than their PE counterparts, with ACsub being the most active, followed by PECsub, ACgen, and PECgen in decreasing order of activity. In HT‐29 cells, the two *C. subternata* extracts were equally active, and more so than the *C. genistoides* extracts, which were also equally active. The HepG2 cells were more susceptible to the aqueous extracts than the HT‐29 cells, but not to the PE extracts (*p* ≥ .05).

### 3.3 Inhibition of cell proliferation

The IC_50_ values for the inhibition of cell proliferation also indicate differences between extracts and the susceptibility of the cells to the extracts (Figure 1). In the HepG2 cells, the PE extracts exhibited higher activity (lower IC_50_ values) than the aqueous extracts, with PECsub being the most active. ACsub and PECgen had the same activity. In HT‐29 cells, both *C. subternata* extracts were equally effective in inhibiting cell proliferation, as well as more effective than the two *C. genistoides* extracts, which were also equally effective. HepG2 cells were again more susceptible than HT‐29 cells to treatment, but only for the PE extracts. The opposite trend was found for ACgen, while ACsub was equally effective in inhibiting cell proliferation in both HepG2 and HT‐29 cell models.

### 3.4 Modulation of apoptosis

**FIGURE 2:**
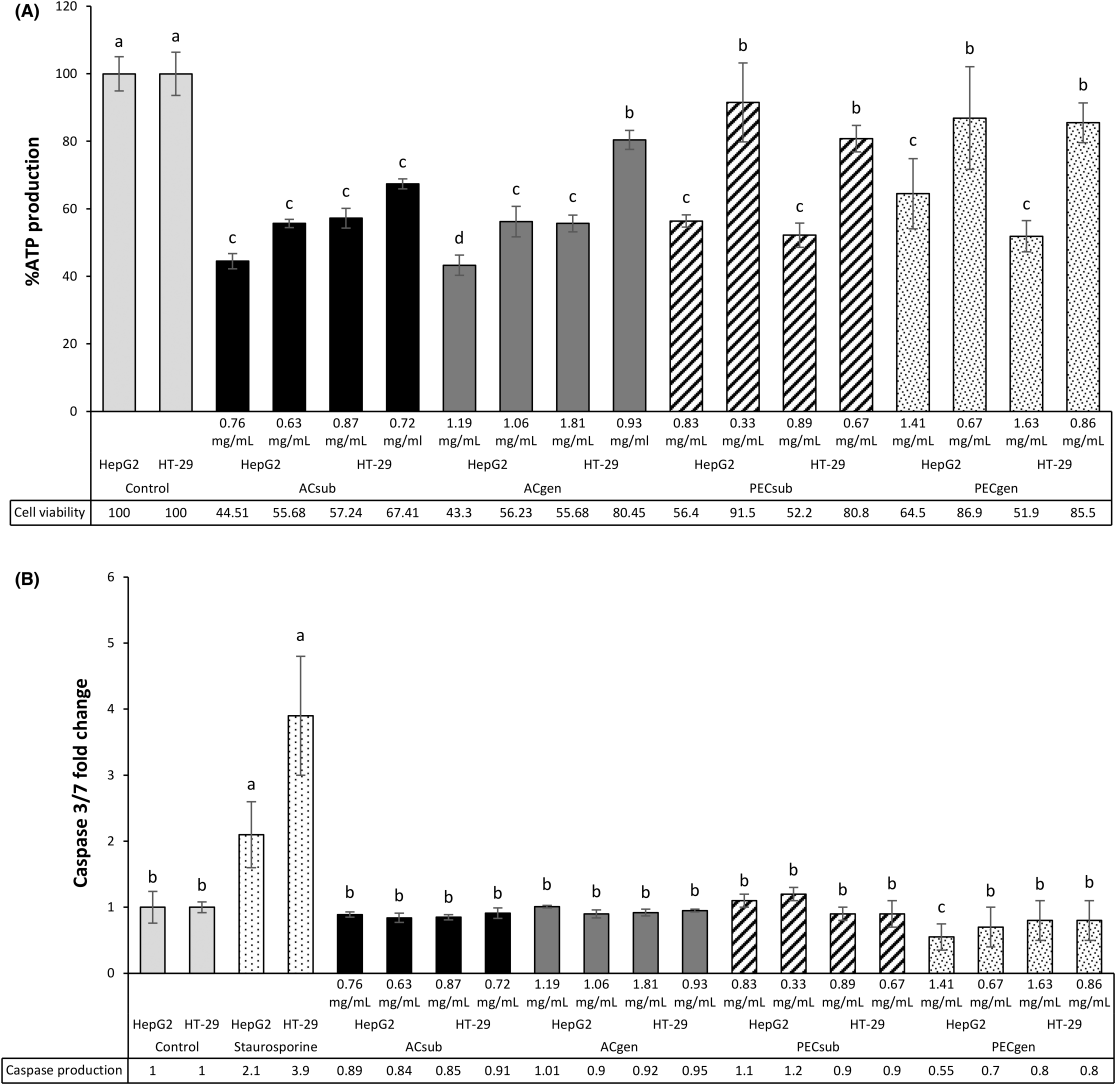
*Cyclopia subternata* and *C. genistoides* extracts were assessed for their effect on (A) cell viability (% ATP production) and (B) caspase production (caspase‐3‐fold difference), using concentrations equivalent to respective IC_50_ values for cell viability and proliferation inhibition in liver cancer cells (HepG2) and colon cancer cells (HT‐29). Mean ± standard deviation values (*n* = 5, from ≥2 experiments) are presented for HepG2 and HT‐29 cells. Independent statistical analysis was performed for each cell line, denoting significant differences (*p* < .05) with distinct lowercase letters. The caspase‐3‐fold increase was calculated against the relative light units of caspase‐3 production in control cells. IC_50_ is the concentration yielding 50% inhibition. ACsub and PECsub refer to the aqueous and polyphenol‐enriched extracts of *C. subternata*, respectively. Similarly, ACgen and PECgen denote the aqueous and polyphenol‐enriched extracts of *C. genistoides*, respectively. ^#^Caspase production analyses were conducted using staurosporine as a positive control.

**FIGURE 3:**
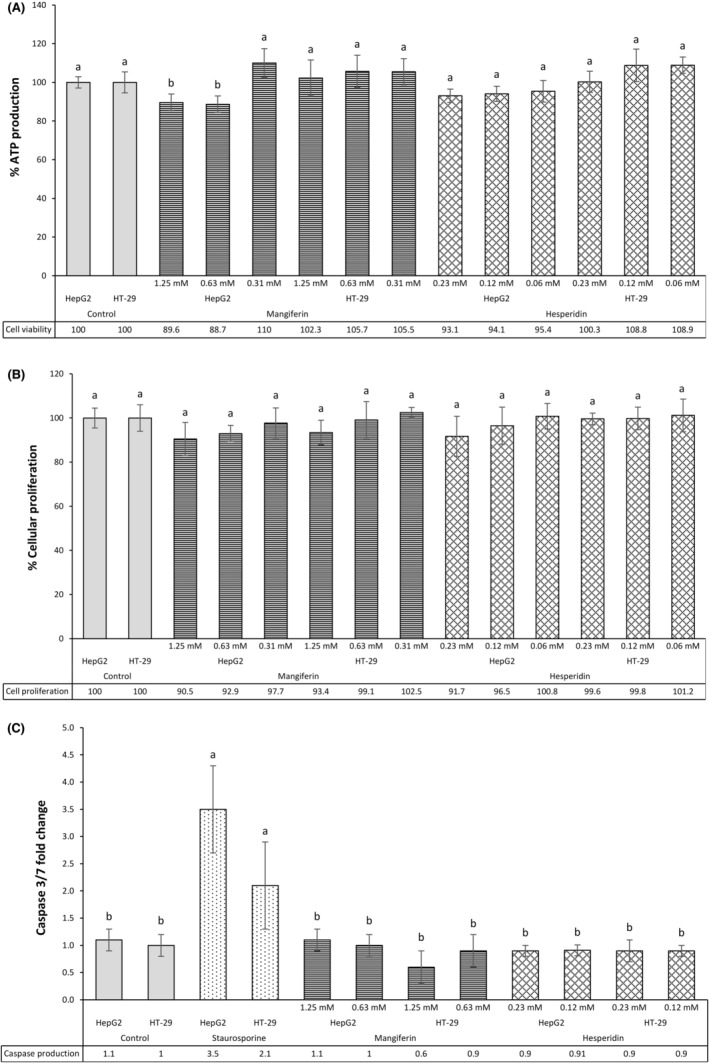
The effects of mangiferin and hesperidin on cell viability (% ATP production) (A), cell proliferation (BrdU incorporation) (B), and caspase production (caspase‐3‐fold increase) (C) by mangiferin and hesperidin, with assays conducted in human liver (HepG2) and colon (HT‐29) cancer cells. Mean ± standard deviation values are presented from five determinations of at least two experiments. Statistical analyses of the different mangiferin and hesperidin concentrations and controls were compared independently for each of the cell cultures. Significant differences (*p* < .05) between means are indicated using distinct lowercase letters. A caspase‐3‐fold increase was calculated against the relative light units of caspase‐3 production in control cells.

None of the honeybush extracts induced apoptosis (Figure 2A) in the cancer cells, even at concentrations reflecting the IC_50_ values for the reduction of cell viability and inhibition of cell proliferation (Figure 2B). Only PECgen significantly (*p* < .05) reduced the baseline apoptosis in HepG2 cells at the highest concentration used. Mangiferin and hesperidin were poorly soluble in the incubation medium, which limited the highest concentrations of the pure compounds that could be tested to 1.25 and 0.23 mM, respectively (Figure 3). Mangiferin reduced (*p* < .05) cell viability (Figure 3A) at the two highest concentrations in HepG2 cells. However, at these concentrations, both polyphenols lacked any effect on cell proliferation (Figure 3B) or apoptosis (Figure 3C). These concentrations were much higher than their equivalent concentrations (mM) based on the IC_50_ values of the aqueous extracts for the reduction of cell viability and inhibition of cell proliferation in HepG2 cells. This was also the case for HT‐29 cells.

### 3.5 Modulation of different types of cell death

**FIGURE 4:**
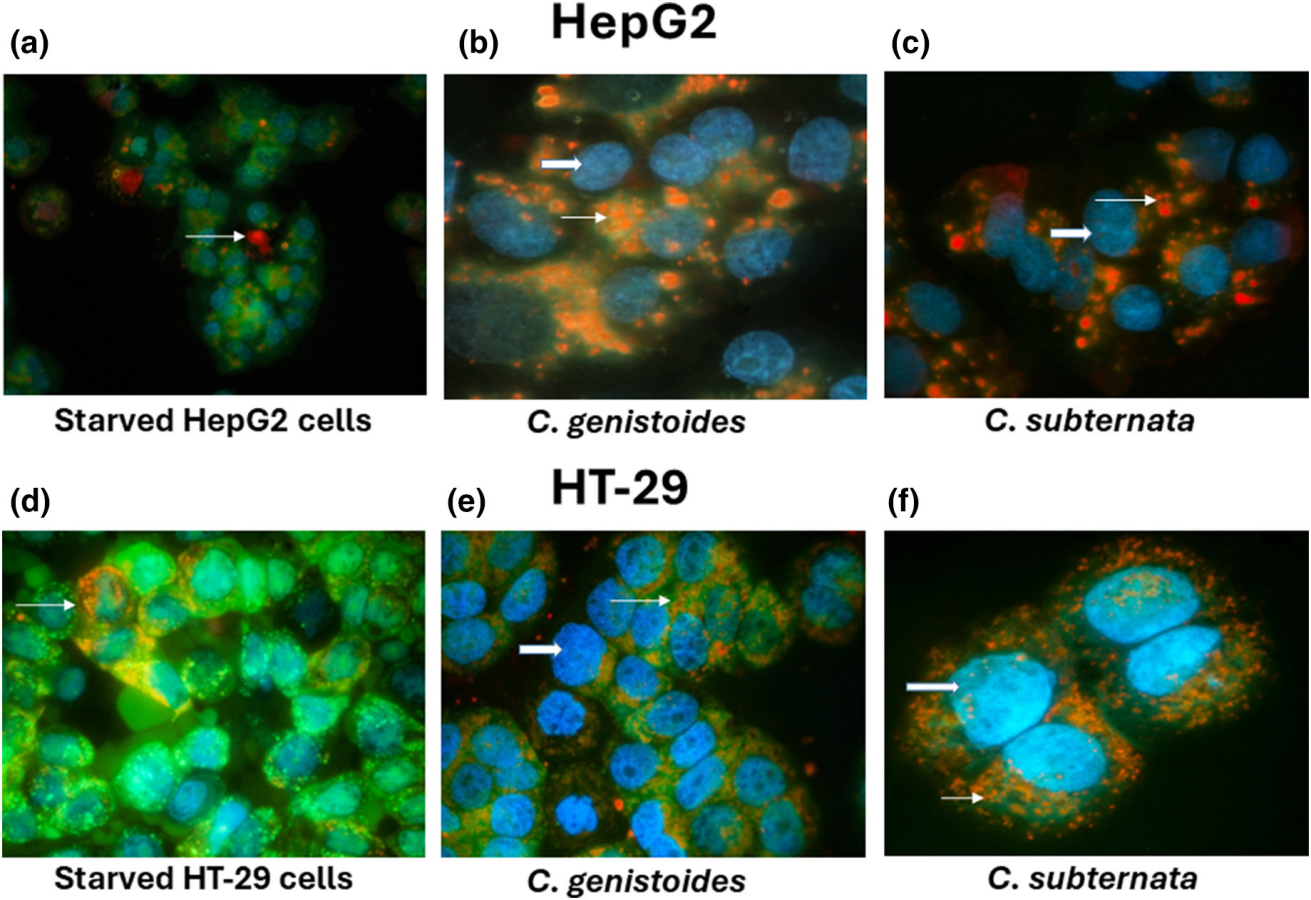
Triple staining with Hoechst 33342 (HO)—apoptosis, acridine orange (AO)—acidic vesicular organelles (AVO), and propidium iodide (PI) (plasma membrane permeability reflecting necrosis) in HepG2 and HT‐29 cells for 24 h. (a) Starved (nutrient‐free conditions) HepG2 cells exhibiting AVO (arrow) merged; (b) and (c) HepG2 cells treated with ACgen (1.06 mg/mL) and ACsub (0.63 mg/mL), respectively, exhibiting brightly stained AVO (arrow) and uniformly stained viable cells with HO (thick arrow); (merged); large punctate spots indicated the presence of AVO, markers for autophagosomes. (d–f) Depict triple staining of HT‐29 starved, ACgen (1.81 mg/mL), and ACsub (0.87 mg/mL) treated HT‐29 cells (magnification ×100). ACsub and PECsub refer to the aqueous and polyphenol‐enriched extracts of *Cyclopia subternata*, respectively. Similarly, ACgen and PECgen denote the aqueous and polyphenol‐enriched extracts of *C. genistoides*, respectively.

The comparative influence of both ACsub and ACgen extracts relative to the PECsub and PECgen extracts demonstrated an augmented decrease in ATP production across both cell lines, as depicted in Figure 2A. Consequently, we opted to conduct further assessments exclusively using the AC extracts. An increase in the presence of acidic vesicular organelles (AVOs) was detected in both cancer cells by AO staining following starvation of HepG2 (Figure 4a) and HT‐29 (Figure 4d) cells in nutrient‐free medium. Cancer cells treated with the aqueous extracts ACgen and ACsub, at their respective IC_50_ concentrations equalling those obtained for the reduction in cell viability, showed a marked induction of AVOs suggestive of the induction of autophagy in HepG2 (Figure 4b,c) and HT‐29 (Figure 4e,f) cells. Both mangiferin and hesperidin lacked any response regarding the induction of AVOs, apoptosis, or necrosis at the concentrations investigated (data not shown).

Triple staining by HO confirmed that the HepG2 and HT‐29 cells treated with the aqueous *Cyclopia* extracts and the two compounds lacked the presence of apoptotic bodies, while no evidence of necrotic cells, reflected by uptake of PI, could be recorded using fluorescence microscopy. Compared to starved cells, more AVOs were observed in cells exposed to *C. genistoides* and *C. subternata*. This could possibly be due to the formation of autolysosomes for bulk degradation of cytoplasmic constituents in response to an autophagy inducer that enhances vesicle acidification beyond what is required during starvation.

**FIGURE 5:**
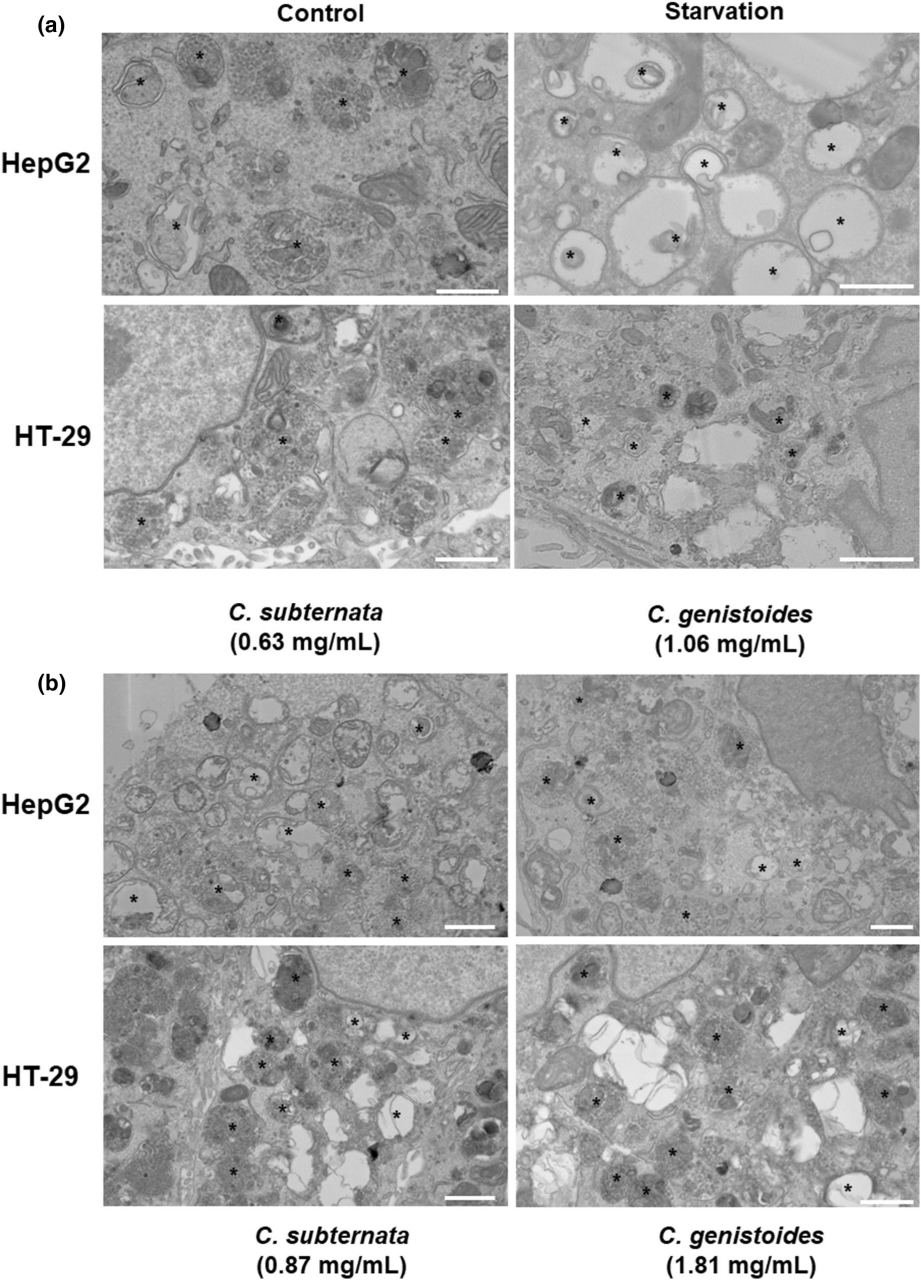
Scanning transmission electron microscopy imaging of HepG2 and HT‐29 cells under (a) control and starvation conditions and (b) in the presence of ACsub and ACgen at IC_50_ concentrations for the reduction in cell viability. IC_50_ is the concentration yielding 50% inhibition. ACsub and ACgen denote the aqueous extracts of *Cyclopia subternata* and *C. genistoides*, respectively. *Autophagic vacuoles. Scale bar: 1 μm.

**FIGURE 6:**
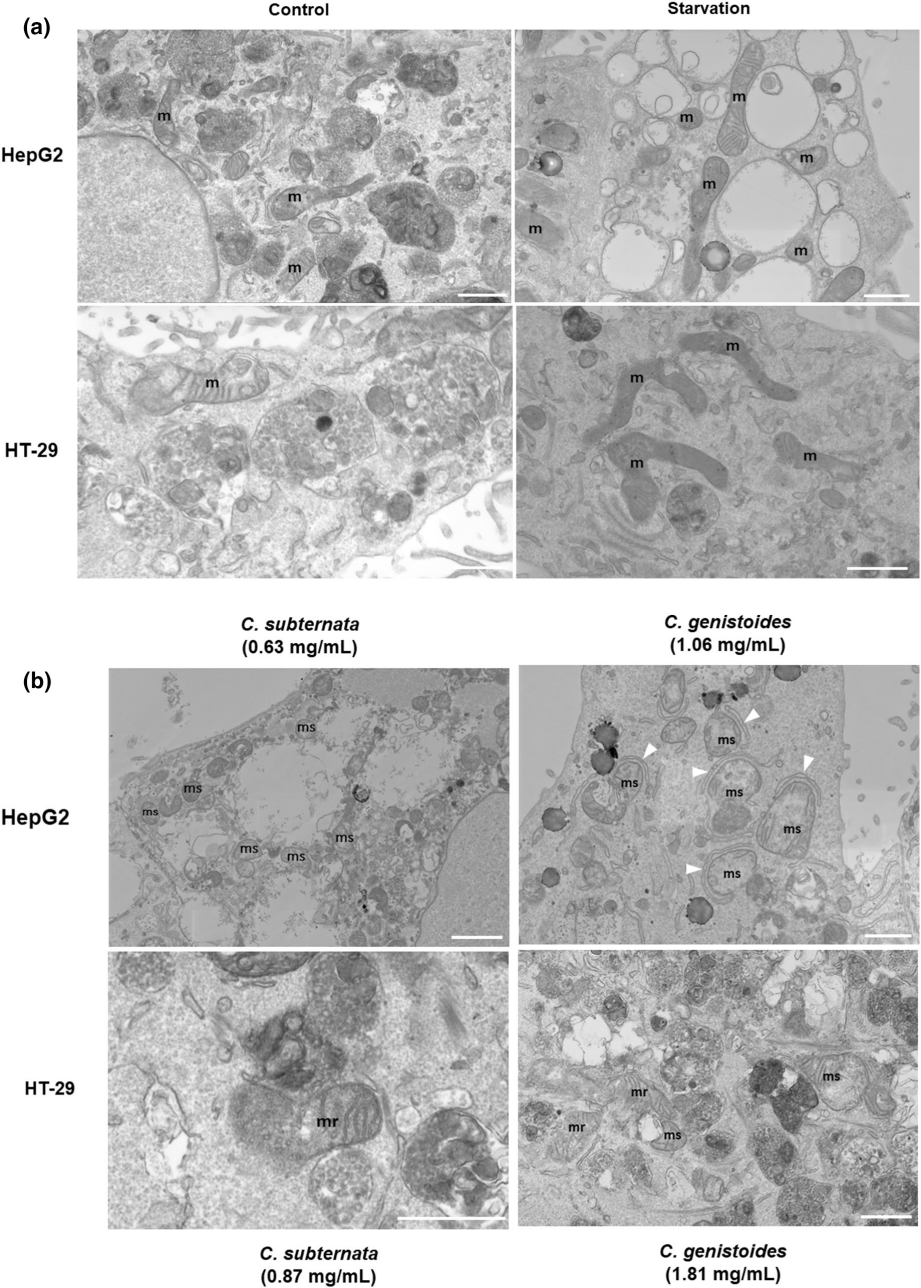
Scanning transmission electron microscopy imaging of HepG2 and HT‐29 cells under control and starvation conditions (a) and after treatment at IC_50_ concentrations for the reduction in cell viability with ACsub and ACgen (b) for 24 h. IC_50_ is the concentration yielding 50% inhibition. ACsub and ACgen denote the aqueous extracts of *Cyclopia subternata* and *C. genistoides*, respectively. arrow heads, phagophores around swollen and damaged mitochondria. m, mitochondria; mr, ruptured mitochondria; ms, swollen mitochondria. Scale bar: 1 μm.

STEM showed that under control conditions, cells presented a healthy cytoplasm with empty and electron‐dense autophagic vacuoles (AVs) visible, indicative of basal levels of autophagy, as well as heterogeneous mitochondrial morphology (Figure 5a). Following starvation, HepG2 cells predominantly displayed empty vacuoles with a distinct double membrane, characteristic of late‐stage AVs with degraded cargo. HT‐29 cells also displayed both empty vacuoles and electron‐dense AVs, indicating a less pronounced autophagy response. In addition to vacuolar changes, both cell lines displayed pronounced changes in mitochondrial morphology (Figure 6a). Mitochondria presented with both elongated and rounded morphologies during both control and starvation conditions, indicating balanced fission and fusion events.

ACsub displayed a pronounced increase in AVs throughout the entire surface area of HepG2 and HT‐29 cells. However, they were more abundant in HepG2 cells, whereas HT‐29 cells presented with both large electron‐dense and empty AVs. ACgen effected an increase in electron‐dense AVs for both HepG2 and HT‐29, characteristic of enhanced cytoplasmic engulfment during the early stages of autophagic degradation (Figure 5b). Empty vacuoles were also present, but not to an overwhelming extent. Both cell lines, therefore, presented with a phenotype characteristic of high autophagic activity to the extent of bulk cytoplasmic vacuoles.

In addition to vacuolar changes, both cell lines displayed pronounced changes in mitochondrial morphology (Figure 6b). Mitochondria presented with both elongated and rounded morphologies during both control and starvation conditions, indicating balanced fission and fusion events. Following treatment with ACsub, the mitochondrial morphology in HepG2 cells was predominantly rounded with reduced cristae, while evidence of ruptured mitochondria was found in HT‐29. ACgen effected predominantly rounded mitochondria with reduced cristae in HepG2 cells. Evidence of mitophagy was also present, with Golgi‐like structures enveloping these potentially damaged mitochondria (Figure 6b). Swollen and rounded mitochondria were also visible in both cell lines indicating extensive mitochondrial damage.

The general cell morphology was inconsistent with that of apoptotic or necrotic cells. The characteristic cell blebbing of apoptosis was not present, and although large vacuoles were observed, the disintegration of organelles was also not observed as would be expected during necrosis.

## 4 DISCUSSION

The various physiological activities of polyphenol compounds may be associated with stabilizing the generation of reactive oxygen species (ROS) and arresting the series of alterations in various cellular, biochemical, and molecular changes associated with carcinogenesis (Pereira et al., 2009). As polyphenols are electron‐rich compounds that contain several hydrolyzable hydroxyl functional groups, they may stabilize themselves when an electron is lost after hydrogen donation and thereby do not become radicals themselves. However, these redox‐sensitive compounds may initiate autoxidation and behave like pro‐oxidants under certain conditions, thereby enhancing the production of ROS (Zhang & Tsao, 2016), disrupting mitochondrial membranes, which could lead to dysfunction and adversely affect cell survival (Galati et al., 2006; Lemarié et al., 2004; Ramos, 2008; Tang et al., 2005). Since mitochondria play a significant role in energy metabolism, ROS production and dysfunctional redox signaling may result in changes that have a biochemical foundation for favorably affecting cancer cells (Chen et al., 2010).

The role of polyphenols in disrupting mitochondrial membrane potential due to their protonophoric‐ and pro‐oxidant‐induced stress responses has been associated with their chemopreventive properties (Stevens et al., 2018). The development of oxidative stress and the start of apoptosis are caused by the disturbance of the mitochondrial redox state through interactions with the electron transport complexes and glutathione (Sandoval‐Acuña et al., 2014; Teixeira et al., 2018). In a previous study, we investigated the differences in anti‐proliferative and pro‐apoptotic effects of green rooibos and green tea aqueous extracts, as well as an aspalathin‐enriched green rooibos extract, in HepG2 and HT‐29 cancer cells. Rooibos flavonoids, aspalathin, and luteolin, and the green tea flavanol, epigallocatechin gallate, were also included to determine their contribution relative to their extract concentrations. This study made it clear that the primary cause of the stimulation of apoptotic cell death is the disturbance of mitochondrial function, as seen by the activation of caspase‐3 in HepG2 cells. Synergistic and/or additive effects between rooibos polyphenolic compounds were shown to be likely responsible for the decrease in cell survival indices and increase in apoptosis, specifically in HepG2 cells (Samodien et al., 2021). It is not known at present whether polyphenol interactions with the electron transport complexes are directly related to their iron‐chelating properties; however, disruption of complex II by the iron chelator, desferrioxamine mesylate, implies that interaction with iron could be critical in the disruption of mitochondrial respiratory function (Yoon et al., 2003).

The current study indicated that *Cyclopia* extracts differentially disrupt cell survival indices in HepG2 and HT‐29 cancer cells, implying disruption of mitochondrial membrane integrity, presumably via the interaction with cellular iron. The two *C. subternata* extracts with the highest FRAP activity were more active in reducing cell viability and inhibiting proliferation in these cancer cells compared to the *C. genistoides* extracts. The differences in the iron‐reducing activity and IC_50_ concentrations of the extracts suggest differential roles of the phenolic compounds. In HepG2 cells, but not HT‐29 cells, the *C. subternata* and *C. genistoides* aqueous extracts were more active than the PE extracts when considering cell viability, while the opposite effect was obtained for cell proliferation. The qualitative and quantitative differences in phenolic composition and the remaining matrices of the extracts most likely determine the activity in disrupting cell growth parameters in cancer cells, but it also depends on the type of cancer cell. The cell type‐dependent effects could be related to differences in the underlying mechanisms that prevail in the disruption of cell viability and inhibition of cell proliferation associated with either the disruption of mitochondrial activity (Stevens et al., 2018) or signaling pathways involving different protein kinases. This also became apparent since PECsub and PECgen were more active in the inhibition of cell proliferation compared to the reduction of cell viability in both cancer cells. A similar trend was noticed for both aqueous extracts in HT‐29 cells; while no difference was noticed for the HepG2 cells treated with ACsub, ACgen was less active in inhibiting cell proliferation.

The high levels of xanthones and benzophenones in the *C. genistoides* extracts compared to the *C. subternata* extracts may explain their relatively high IC_50_ values for reduction of cell viability in HepG2 cells, suggesting protection against the reduction in cell viability. Mangiferin was reported to exhibit anticancer properties (Gold‐Smith et al., 2016). In the present study, mangiferin lacked any effect on cell viability or proliferation in the cancer cells, despite being tested at concentration levels that were far above its equivalent concentration based on the IC_50_ values of the extracts. These results suggest that more than concentration, especially of a single compound, is at play.

The underlying properties of mangiferin are of interest with respect to the redox status of cells, as the compound is known to be an efficient iron chelator with strong radical scavenging ability and antioxidant properties (Matkowski et al., 2013). The high redox potential and formation of a stable iron(III) complex prevent mangiferin from undergoing pro‐oxidation and causing dysfunction in the mitochondria (Pardo‐Andreu et al., 2007). It was shown that a mangiferin‐Fe(III) complex lacks the induction of mitochondrial permeability transition (MPT) by scavenging ROS and also protects mitochondrial membrane protein thiols and glutathione against oxidation. As a result, the anti‐oxidant effects of mangiferin and other catechol‐containing antioxidants are potentially protected by Fe(III) coordination in the context of MPT induction. Mangiferin slightly reduced cell viability in HepG2 cells at the highest concentrations, which could be related to pro‐oxidant effects. However, the interaction of polyphenols with metals is characterized by two competing effects, i.e. metal chelation, which suppresses the catalytic activity of metal ions via the Fenton‐type reaction, and the reduction of metal ions, which results in increased hydroxyl radical formation due to the reducing capacity of polyphenols (Jomová et al., 2019). Mangiferin induces antiproliferative effects in pancreatic carcinoma cells through the generation of endogenous ROS and cell cycle arrest (Yu et al., 2019). Other *Cyclopia* polyphenols can also act as more or less effective antioxidants or pro‐oxidants, depending on molecular structure, and may thus contribute to the different responses monitored. A recent study indicated that hesperidin induces oxidative stress in HepG2 cells and enhances the toxic effect of the chemotherapy drug doxorubicin (Korga et al., 2019). The augmented effect of hesperidin was, however, not related to oxidative stress but to changes in the expression of genes related to the glycolytic pathway. As for mangiferin, no IC_50_ value could be obtained for hesperidin due to its insolubility in the media, and it likely interacts synergistically with the different phenolic substituents present in the extract matrix. The relative concentrations of xanthones, flavanones, dihydrochalcones, flavones, and benzophenones in the *C. subternata* and *C. genistoides* extracts are likely to play an important role in determining their cytotoxic and/or cytoprotective effects against oxidative stress and cell survival indices of cancer cells. At present, very little information is known about the biological activity of the dihydrochalcones and benzophenones present in the extracts. Benzophenones are a large group of compounds that exhibit great biological diversity, including anti‐cancer properties and cytotoxic and pro‐apoptotic effects in different cancer cells (Wu et al., 2014). Dihydrochalcones are also a large group of natural plant polyphenols reported to exhibit anti‐cancer properties (Stompor et al., 2019).

Polyphenols have been reported to have varying impacts on cancer cells (Chen et al., 1998; Han et al., 2009; Mahbub et al., 2013; Seeram et al., 2004). This could have important implications for cancer chemoprevention, as it is known that cancer cells are more susceptible to oxidative stress insults. It can also be concluded that when treating cells with high concentrations of the extracts, reduced ATP levels are insufficient to support apoptosis, as it requires energy to be executed. Studies indicated that defects in the apoptotic signaling pathway result in cells making use of autophagy to remove damaged cells (Chiu et al., 2009; Xie et al., 2011; Xu et al., 2006). The current study provides the first evidence that honeybush extracts elicit autophagy in response to the reduction of cell viability and inhibition of cell proliferation in HepG2 and HT‐29 cells. The reduction in cell viability resulted from a reduction in ATP production due to loss of MPT, mitochondrial swelling, and membrane disruption causing mitophagy, as indicated by the presence of phagophore structures and numerous AVs. Disruption of mitochondrial membrane integrity is known to play a major role in censoring cells to undergo autophagy, with ROS suggested to play a regulatory role in mediating the process (Lee et al., 2012; Roca‐Agujetas et al., 2019). The ROS species, hydroxyl and superoxide anion radicals, and hydrogen peroxide play an important role in different signaling pathways, including autophagy, which is intended to further reduce oxidative damage to sustain cell survival (Scherz‐Shouval & Elazar, 2011). In this regard, the removal of ROS‐producing mitochondria would support cancer survival and cancer promotion under conditions of hypoxia (Rouschop & Wouters, 2009). At present, it is not clear whether the induction of autophagy by the honeybush extracts promotes or protects against cancer cell death, similar to the role of ROS, as redox regulation may either induce cell survival or promote cell death. Recent studies indicated that mangiferin induced autophagy in normal and cancer cells in vitro (Bai et al., 2018; Hou et al., 2018; Yu et al., 2019). However, in the current study, mangiferin lacks any effect on cell growth parameters, the induction of apoptosis and autophagy at the dosage levels utilized, and is likely to play a more protective role in cancer cell death. The combined properties of the different phenolic compounds in the extracts are more likely a major driving force behind the induction of mitochondrial‐induced oxidative stress and autophagy‐induced cell death.

Cancer cells utilize the pro‐survival mechanism of autophagy to promote proliferation and resistance to cell death (Degenhardt et al., 2006). Contrary to promoting survival, autophagy can also determine the fate of the cell by functioning as both a supporter and even an executioner of cell death under certain conditions (Bhat et al., 2018; Denton & Kumar, 2019; Goodall et al., 2016; Kriel & Loos, 2019). Induction of autophagy has previously been shown to induce cell death in apoptosis‐resistant pancreatic and glioblastoma cancer cell lines (Kriel et al., 2018; Pal et al., 2017) and also to facilitate cell death through a specific subtype of autophagy‐dependent cell death referred to as autosis when autophagy is induced via targeted peptides. Therefore, evidence exists for cell death occurring due to the induction of high levels of autophagy. As the current study indicated that the decrease in cell viability and suppression of cell growth were both linked to the stimulation of autophagy, it would appear to be an alternative route for how *C. subternata* and *C. genistoides* extracts exhibit their chemopreventive properties. This was supported by the pronounced increase in AVs and disruption of mitochondrial integrity in response to high concentrations of the extracts, accompanied by mitochondrial damage and network fragmentation. The presence of large empty vacuoles in both cell lines under these conditions suggests uncontrolled cytoplasmic engulfment due to excessive upregulation of autophagy. This also correlates well with our fluorescence data, where an increase in AVOs was found, suggesting that the cargo‐containing vesicles shown in the STEM micrographs are autolysosomes.

However, when there is mitochondrial dysfunction, cells often adapt by exploiting the glycolytic pathway for energy metabolism and become less sensitive to anti‐cancer drugs such as the polyphenols tested. HT‐29 cancer cells have been reported to effectively utilize glycolysis and are resistant to apoptosis via disruption of mitochondrial‐induced oxidative stress (Mojzeš et al., 2020; Semaan et al., 2016; Svihálková‐Sindlerová et al., 2010). The *Cyclopia* extracts also failed to induce necrosis and apoptosis in both cancer cells, with the HT‐29 cancer cells being more resistant to the reduction of cell viability and inhibition of cell proliferation.

## 5 CONCLUSIONS

In this study, the disruption of mitochondrial membrane integrity by various extracts, potentially mediated through polyphenol/iron interactions, is hypothesized to play a role. However, mangiferin and hesperidin showed no discernible effect, suggesting that additional polyphenols and/or intricate compound interactions likely contribute to the disparate cytotoxic and/or cytoprotective effects observed across the extracts. Therefore, further investigation into the underlying mechanisms of the antiproliferative properties of the extracts, particularly in relation to the induction of autophagy‐mediated cell death, is warranted. The induction of autophagy presents an alternative mechanism supporting the reduction in cell growth, albeit it may also serve as a survival mechanism for cancer cells, particularly under hypoxic conditions. Elucidating the mechanism by which *Cyclopia* extracts, particularly their phenolic constituents, modulate proliferation and cell survival is crucial for the development of novel pharmaceutical agents aimed at preventing and treating cancer with minimal adverse effects on normal cells.

## AUTHOR CONTRIBUTIONS

**Sedicka Samodien:** Data curation (equal); formal analysis (equal); investigation (equal); methodology (equal); project administration (equal); writing – original draft (equal). **Maryna de Kock:** Supervision (equal); writing – original draft (supporting). **Elizabeth Joubert:** Investigation (equal); writing – review and editing (equal). **Dalene de Beer:** Investigation (equal); methodology (equal); writing – review and editing (equal). **Jurgen Kriel:** Investigation (equal); writing – review and editing (equal). **Wentzel C. A. Gelderblom:** Conceptualization (equal); formal analysis (equal); funding acquisition (lead); investigation (equal); methodology (equal); project administration (equal); resources (equal); software (equal); supervision (equal); validation (equal); visualization (equal); writing – original draft (equal); writing – review and editing (equal). **Mariska Lilly:** Investigation (equal); methodology (equal); project administration (equal); resources (equal); supervision (equal); validation (equal); visualization (equal); writing – original draft (equal); writing – review and editing (equal).

## CONFLICT OF INTEREST STATEMENT

All authors declare that they have no conflicts of interest.

## ETHICS STATEMENT

This study does not involve any human or animal testing.

## Supporting information


Appendix S1.

## Data Availability

Data are available on request; contact Dr. Mariska Lilly (lillym@cput.ac.za).
