# Supplementary material for: Autophagy‐induced cell death by aqueous and polyphenol‐enriched extracts of honeybush (Cyclopia spp.) in liver and colon cancer cells
Source: Food Sci Nutr. 2024 May 15;12(8):5647–62. doi: 10.1002/fsn3.4214 (PMC11317699; doi:10.1002/fsn3.4214)
Supplement: Supplementary file 1 — Appendix S1. [file FSN3-12-5647-s001.docx]

**SUPPLEMENTARY MATERIAL**

**Autophagy-induced cell death by aqueous and polyphenol-enriched extracts of honeybush (*Cyclopia* spp.) in liver and colon cancer cells**

Sedicka Samodien^1^, Maryna de Kock^2^, Elizabeth Joubert^3,4^, Dalene de Beer^3,4^, Jurgen Kriel^5^, Wentzel C.A. Gelderblom^6^ and Mariska Lilly^1^

^1^Applied Microbial and Health Biotechnology Institute, Cape Peninsula University of Technology, PO Box 1906, Bellville 7535, South Africa; samodeinse@cput.ac.za (S.S.); lillym@cput.ac.za (M.L.)

^2^Department of Medical Bioscience Program, University of Western Cape, Private Bag X17, Bellville 7535, South Africa; mdekock@uwc.ac.za (M.deK.)

^3^Plant Bioactives Group, Post-Harvest & Agro-Processing Technologies, Agricultural Research Council, Infruitec-Nietvoorbij, Private Bag X5026, Stellenbosch 7599, South Africa; JoubertL@arc.agric.za (E.J.), DBeerD@arc.agric.za (D.deB.)

^4^Department of Food Science, Stellenbosch University, Private Bag X1, Matieland, Stellenbosch 7602, South Africa

^5^Central Analytical Facilities, Electron Microscopy Unit, Stellenbosch University, Private Bag X1, Matieland, Stellenbosch 7602, South Africa; jkriel@sun.ac.za (J.K.)

^6^Department of Biochemistry, Stellenbosch University, Private Bag X1, Matieland, Stellenbosch 7602, South Africa; wgelderb@gmail.com (W.G.)

**Correspondence**

Mariska Lilly, Applied Microbial and Health Biotechnology Institute, Cape Peninsula University of Technology, PO Box 1906, Bellville 7535, South Africa.

E-mail: [lillym@cput.ac.za](mailto:lillym@cput.ac.za)

**Table S1.** Characteristics of phenolic compounds identified in *Cyclopia subternata* extracts.

| **Retention time (min)** | **[M-H]^-^** | **Error (ppm)** | **Molecular formula** | **Fragment ions** | **Compound** |
| --- | --- | --- | --- | --- | --- |
| 2.9 | 569.1497 | -1.6 | C_25_H_29_O_15_ | 449, 317, 287*, 167, 125 | 3-β-D-Glucopyranosyl-4-*O*-β-D-glucopyranosyliriflophenone (IDG) |
| 3.0 | 343.1045 | 4.7 | C_15_H_19_O_9_ | 163, 119* | Unknown |
| 3.8 | 423.0922 | -1.2 | C_19_H_19_O_11_ | 303, 193*, 167, 165, 151, 137, 125, 109, 107, 97 | 3-β-D-Glucopyranosylmaclurin (MMG) |
| 6.1 | 407.0961 | -4.2 | C_19_H_19_O_10_ | 287*, 259, 257, 245, 215, 201, 193, 167, 165, 163, 161, 151, 149, 137, 135, 125, 121, 117, 107, 97, 93 | 3-β-D-Glucopyranosyliriflophenone (IMG) |
| 8.6 | 421.0771 | 1.2 | C_19_H_17_O_11_ | 331, 313, 301*, 285, 271, 259 | Mangiferin |
| 9.0 | 421.0758 | -3.1 | C_19_H_17_O_11_ | 331, 313, 301*, 285, 273, 271, 259 | Isomangiferin |
| 9.1 | 593.1461 | 2.2 | C_27_H_29_O_15_ | 503, 472, 383, 353* | Vicenin-2 |
| 11.0 | 579.1714 | 2.8 | C_27_H_31_O_14_ | 459, 433, 313, 271*, 209, 167, 151, 149, 145, 125 | Narirutin |
| 11.2 | 613.1776 | 1.1 | C_27_H_33_O_16_ | 433, 403, 373*, 331, 251, 239, 209 | 3′,5′-Di-β-D-glucopyranosyl-3-hydroxyphloretin (HPDG) |
| 12.0 | 595.1669 | 1 | C_27_H_31_O_15_ | 287, 151*, 135 | Eriocitrin |
| 13.0 | 593.1486 | -3.4 | C_27_H_29_O_15_ | 285* | Scolymoside |
| 13.3 | 597.1791 | -4.7 | C_27_H_33_O_15_ | 417, 387, 357*, 315, 239, 209 | 3′,5′-Di-β-D-glucopyranosylphloretin (PDG) |
| 15.0 | 577.1572 | 2.6 | C_27_H_29_O_14_ | 269* | Isorhoifolin |
| 15.4 | 609.1810 | -1.5 | C_28_H_33_O_15_ | 301* | Hesperidin |

* Indicates base peak ion

**Table S2.** Characteristics of phenolic compounds identified in *Cyclopia genistoides* extracts

| **Retention time (min)** | **[M-H]^-^** | | **Error (ppm)** | **Molecular formula** | **Fragment ions** | **Compound** |
| --- | --- | --- | --- | --- | --- | --- |
| 3.5 | | 585.1443 | -2.2 | C_25_H_29_O_16_ | 465, 385, 355, 333, 303*, 193 | Maclurin-di-*O,C*-hexose |
| 5.0 | | 343.1038 | 2.6 | C_15_H_19_O_9_ | 163, 119*, 89 | Unknown |
| 6.2 | | 569.1506 | 0 | C_25_H_29_O_15_ | 479, 449, 317, 287*, 167, 125 | 3-β-D-Glucopyranosyl-4-*O*-β-D-glucopyranosyliriflophenone (IDG) |
| 7.1 | | 423.0922 | -1.2 | C_19_H_19_O_11_ | 333, 303, 259, 223, 193*, 165 | 3-β-D-Glucopyranosylmaclurin (MMG) |
| 10.5 | | 285.0611 | 0.4 | C_12_H_13_O_8_ | 153, 109* | dihydroxybenzoic acid-O-pentose |
| 12.5 | | 407.0970 | -2 | C_19_H_19_O_10_ | 317, 287*, 245, 201, 193, 165, 161, 125, 117 | 3-β-D-Glucopyranosyliriflophenone (IMG) |
| 19.7 | | 595.1644 | -3.2 | C_27_H_31_O_15_ | 475, 415, 385, 355*, 313 | Eriodictyol-*O*-deoxyhexose-*O*-hexose (EDH) |
| 23.9 | | 421.0768 | -0.7 | C_19_H_17_O_11_ | 331, 301*, 271, 259 | Mangiferin |
| 24.5 | | 421.0780 | 2.1 | C_19_H_17_O_11_ | 331, 303*, 271, 259 | Isomangiferin |
| 26.4 | | 593.1478 | -4.7 | C_27_H_29_O_15_ | 503, 473*, 425, 383, 353 | Vicenin-2 |
| 27.9 | | 579.1721 | 1.2 | C_27_H_31_O_14_ | 433, 433, 313, 271*, 145, 125 | (2*R*)-5-*O*-neohesperidosylnaringenin (2RNAR)^a^ |
| 29 | | 579.1738 | 4.1 | C_27_H_31_O_14_ | 459, 433, 313, 271*, 151, 145, 125 | (2*S*)-5-*O*-neohesperidosylnaringenin (2SNAR) |
| 32.4 | | 595.1661 | -0.3 | C_27_H_31_O_15_ | 287*, 151, 135 | Eriocitrin |
| 32.8 | | 613.1743 | -4.2 | C_27_H_33_O_16_ | 493, 475, 433, 403, 373*, 331 | 3′,5′-Di-β-D-glucopyranosyl-3-hydroxyphloretin (HPDG) |
| 38.2 | | 593.1503 | -0.5 | C_27_H_29_O_15_ | 285* | Scolymoside^b^ |
| 38.5 | | 597.1804 | -2.5 | C_27_H_33_O_15_ | 477, 459, 417, 387, 157*, 345, 315, 209 | 3′,5′-Di-β-D-glucopyranosylphloretin (PDG) |
| 42.7 | | 609.1825 | 1 | C_28_H_33_O_15_ | 303* | Hesperidin |

* Indicates base peak ion

^a^ Only detected in ACgen; ^b^ Only detected in PECgen.
